# Pyocyanin produced by *Pseudomonas aeruginosa* creates legacy effects that promote antibiotic resistance evolution in enterococci

**DOI:** 10.1128/aac.00078-26

**Published:** 2026-06-22

**Authors:** M. G. J. de Vos, V. Jansen, O. El Bouhlali, A. A. Vlasblom, L. E. Zandbergen, I. van der Windt, C. I. Sey, J. Kool, R. Nijland, A. de Jong, O. P. Kuipers, S. Dunn, A. McNally, J. A. G. M de Visser

**Affiliations:** 1 Groningen Institute for Evolutionary Life Sciences, University of Groningen, Groningen, the Netherlands; 2 Department of Physiology, Amsterdam UMC 685893 , Amsterdam, the Netherlands; 3 Laboratory of Genetics, Wageningen University, Wageningen, the Netherlands; 4 University of Utrecht, Population Health 8125, Utrecht, the Netherlands,; 5 Field Technology Innovations, Wageningen Research, Wageningen, the Netherlands; 6 Marine Animal Ecology, Wageningen University 685467 , Wageningen, the Netherlands; 7 Groningen Biomolecular Sciences and Biotechnology, University of Groningen, Groningen, the Netherlands; 8 School of Infection, University of Birmingham 152871, Birmingham, United Kingdom; University of Fribourg, Fribourg, Switzerland

**Keywords:** antibiotic resistance, secondary metabolites, evolvability, efflux, infectious diseases, microbial ecology

## Abstract

Polymicrobial infections are small communities of multiple interacting bacterial species. Interactions among constituent species may modify the growth of community members in the presence of antibiotics, for example, via degradation of the antibiotic, the induction of specific resistance mechanisms, or increased mutation supply rates. However, for most polymicrobial infections, the nature of such interactions is opaque, while they may affect both treatment efficacy and the evolution of antibiotic resistance. Here, we describe that past interaction of enterococci with *Pseudomonas aeruginosa* creates legacy effects that substantially enhance their antibiotic tolerance and promote resistance evolution. Specifically, we find that the temporary exposure to pyocyanin, a secondary metabolite produced by *P. aeruginosa*, increases antibiotic efflux in enterococci. We show that these tolerance legacy effects enhance the tolerance to rifampicin and nalidixic acid, and thereby promote the evolution of antibiotic resistance of enterococci. This work shows that transient interactions in polymicrobial communities can alter the evolutionary fate of community members.

## INTRODUCTION

Bacterial infections may consist of different species that together form a polymicrobial community, as is the case in urinary tract infections (UTIs) (1–3). The response to antibiotic treatment of such polymicrobial infections may be affected by metabolic interactions among community members that affect the survival and resistance evolution of specific members (4–7). For example, β-lactamase-producing strains may cross-protect community members against β-lactams (8, 9), and antibiotic-resistant *Escherichia coli* strains producing the signal molecule indole may induce antibiotic tolerance via enhanced efflux in susceptible bacteria (10). Previous research of polymicrobial UTIs has shown that interactions between UTI members can alter the growth, antibiotic tolerance, and evolvability of antibiotic resistance of other community members (7, 11, 12). Yet, the nature of interactions affecting the evolvability of antibiotic resistance is often unknown.

The role of Gram-positive bacteria in infections has long been debated, but evidence supporting the importance of these species in polymicrobial UTI ecosystems and other infections is growing (11, 13–19). For long, urine was thought to be sterile in the absence of a UTI. However, based on results from DNA sequencing studies and enhanced culture methods, it is currently clear that a diverse microbial community, including Gram-positive species, is present in the urinary tract. This community is called the urobiome (3, 18, 20–24). The impact of the urobiome on the growth of community members (11, 17), pathogenesis (15, 20), and treatment efficacy of UTIs by antibiotics is currently under investigation. Moreover, the increased emergence and spread of antimicrobial resistance in urobiome-associated species (21–23) necessitates investigation of their evolvability of antimicrobial resistance.

Antibiotic resistance is determined by the genetic constituency of isolates. Yet, the microbial environment can also alter the susceptibility (11, 24–29) to antibiotics. For example, changes in pH (11) or metabolic composition of the environment may alter the susceptibility to antibiotics (30, 31). Such changes in antibiotic tolerance are often associated with reduced growth, which has been implied in persistence (5, 28, 32, 33), but also with improved growth (11).

Effects on the susceptibility to antimicrobials mediated by microbial interactions are manifold. Population sizes, with immediate, density-dependent survival benefits (i.e., the inoculum effect), for instance, due to accelerated antibiotic breakdown (34–36) and other protective population effects (37), can alter antibiotic susceptibility. In addition, the susceptibility level can be affected by metabolic interactions and signal molecules between community members (29). Such microbial interactions between UTI bacteria and in other bacterial co-cultures have recently been shown to alter the course of antibiotic resistance evolution (7, 38–40).

Here, we investigate the effect of bacterial metabolic interactions, with a panel of species that are frequently isolated together in persons diagnosed with polymicrobial UTIs (1), on the evolvability of antimicrobial resistance in enterococci.

## RESULTS

### *P. aeruginosa* increases the production of antibiotic-resistant colonies in *E. faecium*

To test for cross-species interactions that would enhance antibiotic resistance in *Enterococcus faecium*—either through increased mutation rates, antibiotic tolerance, or both—we investigated effects on the production of antibiotic-resistant colonies in *E. faecium* from exposure to conditioned medium produced by *Escherichia coli*, *Klebsiella pneumoniae*, *Proteus mirabilis*, *Pseudomonas aeruginosa*, and *Staphylococcus aureus*, species that often co-occur in polymicrobial UTIs (11). In the polymicrobial four-species UTI communities obtained from urine of elderly persons diagnosed with UTI (1, 11, 41), Enterococcus species co-occur with *E. coli*, *K. pneumoniae*, *P. mirabilis*, *P. aeruginosa*, and *S. aureus* in 70%, 8%, 30%, 30%, and 13% of the communities, respectively. Enterococcus species were present in 80% of those communities. Details of the antibiotic resistance evolvability assay are described in the "Materials and Methods" section. Briefly, conditioned medium was generated by growing single isolates for 48 h in liquid LB medium, recovering the supernatant, and replenishing it with nutrients. To screen for the differential evolution of resistance of *E. faecium* under the different conditioned media, we adapted the P_0_ method (42, 43). We exposed 288 replicate 200 µL populations of *E. faecium* overnight to either conditioned medium of bacterial isolates or LB reference medium, and spotted 8 µL samples of each replicate overnight culture onto fresh LB agar plates (without conditioned medium) containing the antibiotic rifampicin (15 µg/mL, twice its minimal inhibitory concentration, MIC). Based on the number of replicate cultures that showed at least one antibiotic resistant colony, we calculated the evolvability (Materials and Methods). We define the relative evolvability of antibiotic resistance in *E. faecium*, A, as the evolvability of antibiotic resistance in *E. faecium* exposed to conditioned versus unconditioned reference medium (LB): A = *a*_conditioned medium_*/a*_reference medium_. This method allows us to investigate the generation of resistant mutants in the different conditions in the absence of selection for antibiotic resistance. Increases in evolvability of antibiotic resistance (A) in *E. faecium* may be due to effects of conditioned media on the mutation rate, on antibiotic tolerance, or both.

**Fig 1:**
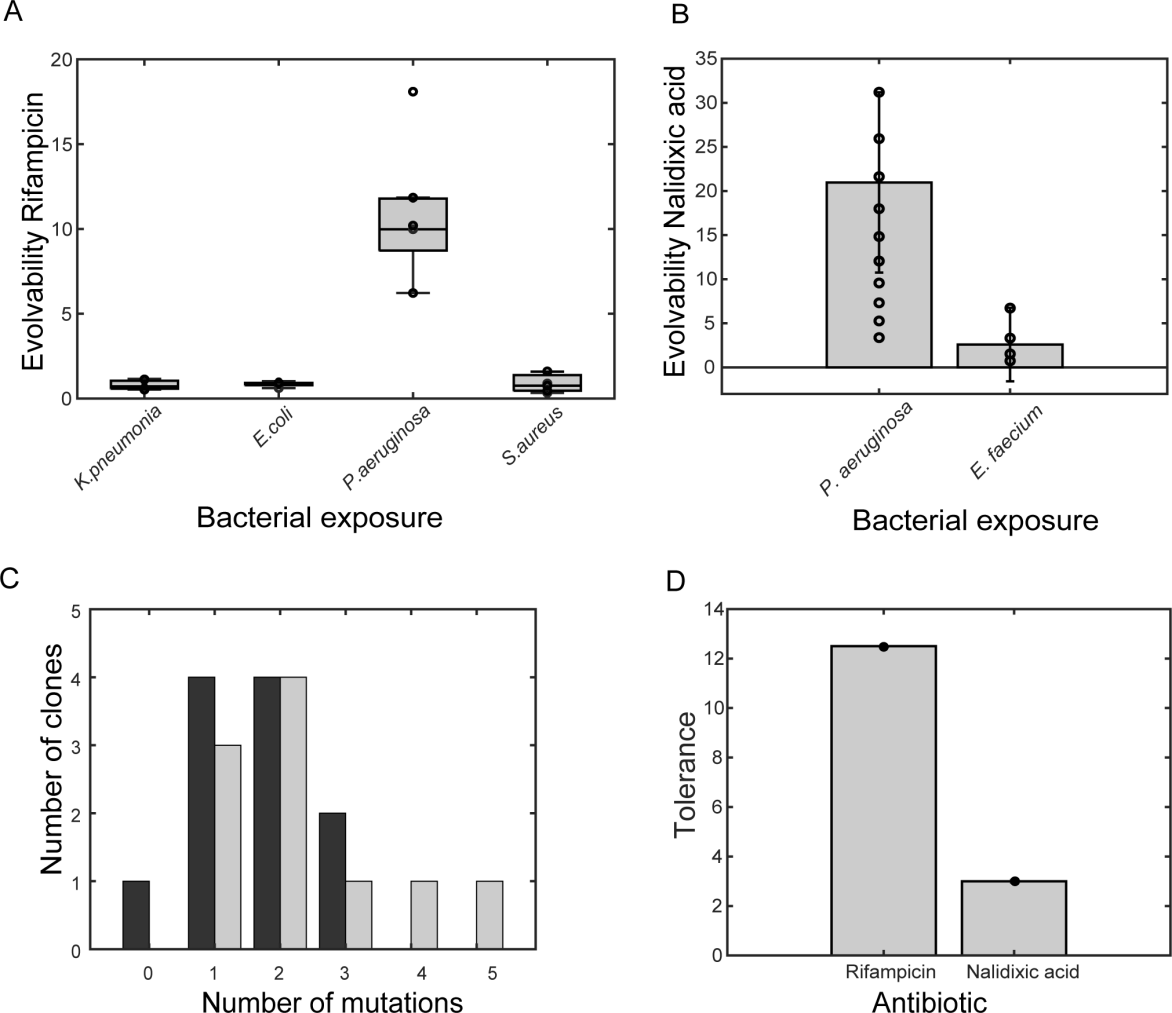
Evolvability of antibiotic resistance mediated by exposure to *P. aeruginosa.* (**A**) Evolvability of rifampicin resistance of *E. faecium* under the influence of conditioned media derived from different bacterial species. Evolvability of rifampicin resistance is measured as the rate of rifampicin resistance in conditioned medium normalized to the rate of rifampicin resistance in LB control medium, as determined by the evolvability assay (Materials and Methods). The presence of the conditioned medium of *P. aeruginosa* increases the evolvability of rifampicin resistance of *E. faecium* (one-way ANOVA, *P* = 4.6e-22, post-hoc Dunnett’s test *P* = 1.2e-06 for *P. aeruginosa medium, N* = 6 for each condition). Other conditioned media of *K. pneumoniae*, *E. coli,* and *S. aureus* do not alter the evolvability of rifampicin resistance. The lines in the box plots represent the median; bottom and top edges represent the lower and upper quartiles; whiskers represent the end points of the data set; and small circles represent outliers. (**B**) Increased evolvability of nalidixic resistance of *E. faecium* under the influence of *P. aeruginosa*-conditioned medium. Evolvability assay with nalidixic acid instead of rifampicin (Materials and Methods) showed that *P. aeruginosa*-conditioned medium also leads to an increased nalidixic acid resistance evolvability, whereas conditioned medium of *E. faecium* does not (one-way ANOVA, *P* = 0.0005; Dunnett’s post-hoc test, *P* = 0.0005 for *P. aeruginosa*-conditioned medium*, P* = 0.96, *N* = 15, for *E. faecium* conditioned medium, *N* = 8). Bar graphs show average evolvability with standard deviation. (**C**) Distribution of the number of mutations in the resistant clones generated after exposure to *P. aeruginosa*-conditioned medium. The number of mutations in rifampicin-resistant clones generated in the evolvability assay. We sequenced 10 rifampicin-resistant clones of *E. faecium*-generated after transient exposure to *P. aeruginosa*-conditioned medium (light bars) and 11 rifampicin-resistant clones of *E. faecium*-generated after transient exposure to LB controls (black bars). Clones exposed overnight to *P. aeruginosa*-conditioned medium have on average more mutations, compared to LB controls (black bars), yet the distributions are not statistically significant (students *t*-test *P* = 0.09). (**D**) *P. aeruginosa*-conditioned medium mediates antibiotic tolerance. *P. aeruginosa*-conditioned medium induces antibiotic tolerance of rifampicin and nalidixic acid in *E. faecium*. Bar graphs show the fold increase in tolerance compared to LB reference medium (Materials and Methods), with error bars depicting the standard deviation of the measurement (*N* = 3).

We find that the transient, overnight, exposure to most conditioned media (i.e., from *E. coli*, *K. pneumoniae*, and *S. aureus*) does not affect the evolvability of rifampicin resistance of *E. faecium*. However, there is one notable exception: the presence of the conditioned medium of *P. aeruginosa* increases evolvability ~10 times compared to the populations that were not exposed to this conditioned medium (One-way ANOVA, *P* < 0.01, post-hoc Dunnett’s test *P* < 0.01, Fig. 1A). Overnight regrowth of single colonies that appeared in the evolvability assay in LB (in the absence of antibiotics), followed by plating on LB agar plates containing 50 µg/mL rifampicin, showed that colonies were highly resistant (over 50 µg/mL rifampicin) and that resistance was heritable (Materials and Methods).

We know that conditioned media can alter the nutrient concentration and the constituents of the medium. We found no effect of the nutrient concentration on evolvability of resistance (Fig. S1A). Testing a larger set of bacterial interactions via conditioned media (i.e., from two *E. coli* isolates, two *P. mirabilis* isolates, and single isolates of *S. haemolyticus*, *E. faecium,* and *E. faecalis*), we find that the magnitude of the effect on evolvability of *E. faecium* is not related to population size as a consequence of the nutrient status of the conditioned medium (Fig. S1B), in contrast to findings of Krašovec et al. (44). This also shows that *E. faecium* can grow well in the presence of most conditioned media; additionally, they can grow in co-culture with *P. aeruginosa* (Table S1). Conditioned medium can also lower the general efficacy of antibiotics via other changes to the medium related to nutrient composition, for instance, by increasing the pH (11). If the conditioned medium of *P. aeruginosa* had such an efficacy-lowering effect on rifampicin, we would expect this to also affect other species than *E. faecium*. Therefore, to rule out that *P. aeruginosa*-conditioned medium lowered the general efficacy of rifampicin; we performed the antibiotic resistance evolvability assay with two other focal species, *E. coli* and *K. pneumoniae. E. coli* and *K. pneumoniae* did not experience an increased phenotypic evolvability in the presence of *P. aeruginosa*-conditioned medium (one-way ANOVA, *P* = 0.25, Table S2). We can, therefore, conclude that the increased evolvability of rifampicin resistance in *E. faecium* due to *P. aeruginosa*-conditioned medium is not an artifact caused by the decreased efficacy of rifampicin.

To test the generality of the increased antibiotic resistance evolvability of *E. faecium* after transient exposure to *P. aeruginosa*-conditioned medium, we performed a similar evolvability assay with nalidixic acid instead of rifampicin. Nalidixic acid has another mode of action than rifampicin: while rifampicin mainly inhibits RpoB, the β subunit of the RNA polymerase, nalidixic acid inhibits DNA gyrase activity (45). We find that the transient exposure to *P. aeruginosa*-conditioned medium also leads to the increased evolvability of nalidixic acid resistance of *E. faecium*, whereas the conditioned medium of *E. faecium* itself does not have this effect (one-way ANOVA, *P* = 0.0005, post-hoc Dunnett’s test, *P* = 0.0005 for *P. aeruginosa*-conditioned medium, *P* = 0.96 for *E. faecium* conditioned medium; Fig. 1B). These combined results suggest a particular interaction of *P. aeruginosa* and *E. faecium* on the evolvability of antibiotic resistance.

### Genetic basis of resistance

To test whether the increased evolvability of *E. faecium* after transient exposure to *P. aeruginosa*-conditioned medium corresponded to an increased genomic mutation rate, we sequenced the genomes of evolved isolates derived from the phenotypic evolvability assay. Sequencing random clones with and without transient exposure to *P. aeruginosa* would more directly allow us to test the expected 10-fold increase in mutation rate, but is practically not feasible, as it would require sequencing many more clones, assuming a wild-type genomic mutation rate in the order of 0.003 per generation (46). We expect all rifampicin-resistant clones to have at least one mutation, and only a slightly higher mutation frequency if the mutation rate would be 10-fold higher. For example, for a mutation rate of 0.003 mutations/genome/generation, the Poisson probability for rifampicin-resistant mutants having more than one mutation is ~0.15%, while this increases to only ~1.5% for a 10-fold higher mutation rate.

By means of whole-genome sequencing of highly resistant (>50 µg/mL rifampicin) mutants that initially appeared on the rifampicin (15 µg/mL) containing agar plates (Materials and Methods), we found mutation frequencies that were inconsistent with these expectations: one of the control clones had no mutations, while the majority had multiple mutations (Fig. 1C). The fact that also approximately 70% of the control clones contain more than one mutation suggests that mutations also arise during colony growth on the rifampicin-containing plates. However, clones derived after the transient exposure to *P. aeruginosa*-conditioned medium had, on average, 40% more, as well as different, mutations compared to those exposed to LB (Table S3 and S4 and Fig. 1C), although the difference in mutation frequency was not statistically significant (Student’s *t*-test, two-tailed *P* = 0.09). Interestingly, clones exposed to *P. aeruginosa*-conditioned medium contained more mutations outside the target *rpoB*, i.e., 78% versus 50% for the control clones (Fisher’s exact *P* = 0.0464), with control clones showing canonical rifampicin resistance *rpoB* mutations (47), whereas the clones exposed to *P. aeruginosa*-conditioned medium mostly contained different *rpoB* mutations (Table S3). Mutations outside *rpoB* in clones exposed to *P. aeruginosa* included mutations in the multidrug resistance protein *stp* (48), and *pyrF* (49) and *nrnA* (50), which are involved in the production and degradation of nucleotides (51) (Table S4).

We investigated whether SOS or stringent response genes, which are associated with increased mutation rates (52–55), were upregulated after the exposure to *P. aeruginosa*-conditioned medium. We did not find any evidence for the upregulation of these stress response genes. Yet, the transcriptomic analyses did reveal a downregulation in *P. aeruginosa* relative to *E. faecium*-conditioned medium of *Dps* (DNA protection during starvation protein, log_2_ fold change −2.73, FDR 8.78e-3, *P* = 5.73e-5), which is involved in oxidative stress protection and has ferroxidase activity, and *Usp* (56) (universal stress protein, log_2_ fold change −2.60, FDR 9.47e-4, *P* = 2.20e-5), involved in general stress protection. The 40% increase in mutation frequency, together with the more diverse nature of mutation targets in clones exposed to *P. aeruginosa*-conditioned medium, therefore suggests that increased antibiotic tolerance allowed for colony growth on the rifampicin plates, which promoted the selection of resistance mutations.

### *P. aeruginosa* induces lasting antibiotic tolerance

To investigate whether the observed frequency increase of resistant colonies in our assay is (partly) due to the physiological induction of certain tolerance mechanisms, we performed an antibiotic tolerance assay. We grew *E. faecium* in a two-fold dilution gradient of rifampicin and nalidixic acid in the presence and absence of *P. aeruginosa*-conditioned medium. We find that *P. aeruginosa*-conditioned medium indeed increases the tolerance to these antibiotics (Fig. 1D), by allowing *E. faecium* to grow at higher concentrations of antibiotic compared to in LB reference medium. This antibiotic tolerance effect—the decreased susceptibility to antibiotics—is in line with the previously observed induced tolerance of enterococci to nitrofurantoin and sulfamethoxazole-trimethoprim combination (11), as well as to vancomycin (7), in the presence of *P. aeruginosa*.

To investigate whether the effect of the transient overnight exposure to *P. aeruginosa*-conditioned medium that we describe above is short-lived, we repeated the evolvability assay with two different additional washing steps before spotting the cultures on the rifampicin-containing LB agar plates. Specifically, we washed a subset of the overnight cultures exposed to *P. aeruginosa*-conditioned medium in PBS before spotting the replicate cultures on the rifampicin-containing LB agar plate. Additionally, we washed a subset of overnight-exposed cultures in LB followed by a 3-hour regrowth in LB (in the absence of conditioned medium), before spotting samples on the rifampicin-containing agar plates. Neither washing with PBS nor a three-hour regrowth in fresh LB ameliorated the increased phenotypic evolvability due to the exposure to *P. aeruginosa*-conditioned medium (Fig. S2). This shows that the increased evolvability induced by *P. aeruginosa*-conditioned medium is rather long lasting.

### *P. aeruginosa* increases antibiotic resistance in enterococci

**Fig 2:**
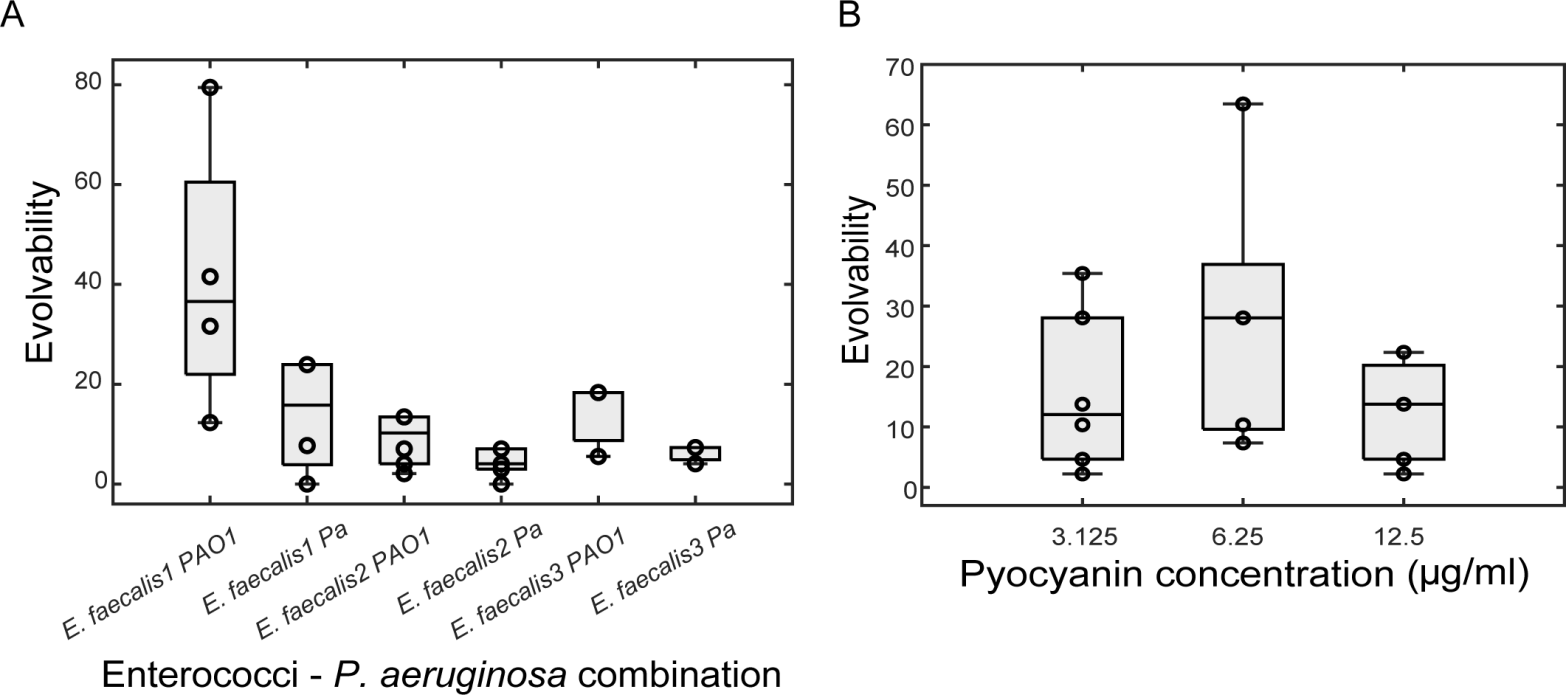
General effect of exposure to *P. aeruginosa*-conditioned medium on antibiotic resistance evolvability of enterococci. (**A**) The evolvability of three different *E. faecalis* isolates (1–3) exposed to PAO1 (canonical *P. aeruginosa*) and uropathogenic *P. aeruginosa* (Pa, used for all experiments described herein) conditioned medium, as measured via the rifampicin evolvability assay (Materials and Methods). The three *E. faecalis* isolates experienced an even higher evolvability after the transient exposure to PAO1 compared to the exposure to uropathogenic *P. aeruginosa* medium (Student’s *t*-test, *P* < 0.009, including Bonferroni-correction for multiple comparisons; *N* = 4, 4, 6, 6, 3, 3, for each respective condition tested). Evolvability of rifampicin resistance is measured as the rate of rifampicin resistance in conditioned medium normalized to the rate of rifampicin resistance in LB reference control medium. The lines in the box plots represent the median; bottom and top edges represent the lower and upper quartiles; whiskers represent the end points of the data set. (**B**) Pyocyanin leads to increased evolvability of rifampicin resistance in *E. faecium*. Different physiologically relevant concentrations of pyocyanin (3.125, 6.25, 12.5 µg/mL) in LB lead to the increased evolvability of rifampicin resistance, as determined by the evolvability assay, normalized to the rate of rifampicin resistance in LB reference control medium (one-way ANOVA, *P* = 0.01, post-hoc Dunnett’s test, *P* = 0.04 for 3.125 mg/mL, *P* = 0.01 6.25 mg/mL and *P* = 0.4 12.5 mg/mL pyocyanin, *N* = 6 for each condition). The lines in the box plots represent the median; bottom and top edges represent the lower and upper quartiles; whiskers represent the end points of the data set.

To investigate whether other isolates of *P. aeruginosa* and other enterococci also experienced increased phenotypic antibiotic resistance, we tested the effect of canonical *P. aeruginosa* isolate PAO1 and the uropathogenic *P. aeruginosa* isolate used above on three uropathogenic *E. faecalis* strains from our collection (1). These followed a similar pattern, with some combinations showing an even higher elevated phenotypic evolvability of resistance, induced by the transient overnight exposure to *P. aeruginosa*conditioned medium (Fig. 2A). This suggests that the increased evolvability is not limited to these initially tested isolates but is rather due to a general *P. aeruginosa* – enterococci interaction.

### Pyocyanin produced by *P. aeruginosa* induces increased efflux in enterococci

We noted that, in particular, the PAO1-conditioned medium with a green tint, but not the un-tinted PAO1-conditioned medium or untinted PAO1 quorum-sensing-mutant conditioned medium generated under lower oxygen conditions, led to a higher number of resistant colonies of *E. faecium* (Fig. S3A). This led us to hypothesize that pyocyanin, which is responsible for the colored hue in the conditioned medium, is produced under aerobic conditions and plays an important role in the virulence of *P. aeruginosa* (57, 58), causes the increased numbers of rifampicin-resistant colonies in the evolvability assay. Testing the effect of overnight exposure to physiologically relevant concentrations of pyocyanin (59) on the evolvability of rifampicin resistance indeed showed an increase of rifampicin-resistant colonies (Fig. 2B). Phenazine, a structurally similar compound and precursor of pyocyanin, did not lead to an increase in rifampicin resistance (Fig. S3B).

**Fig 3:**
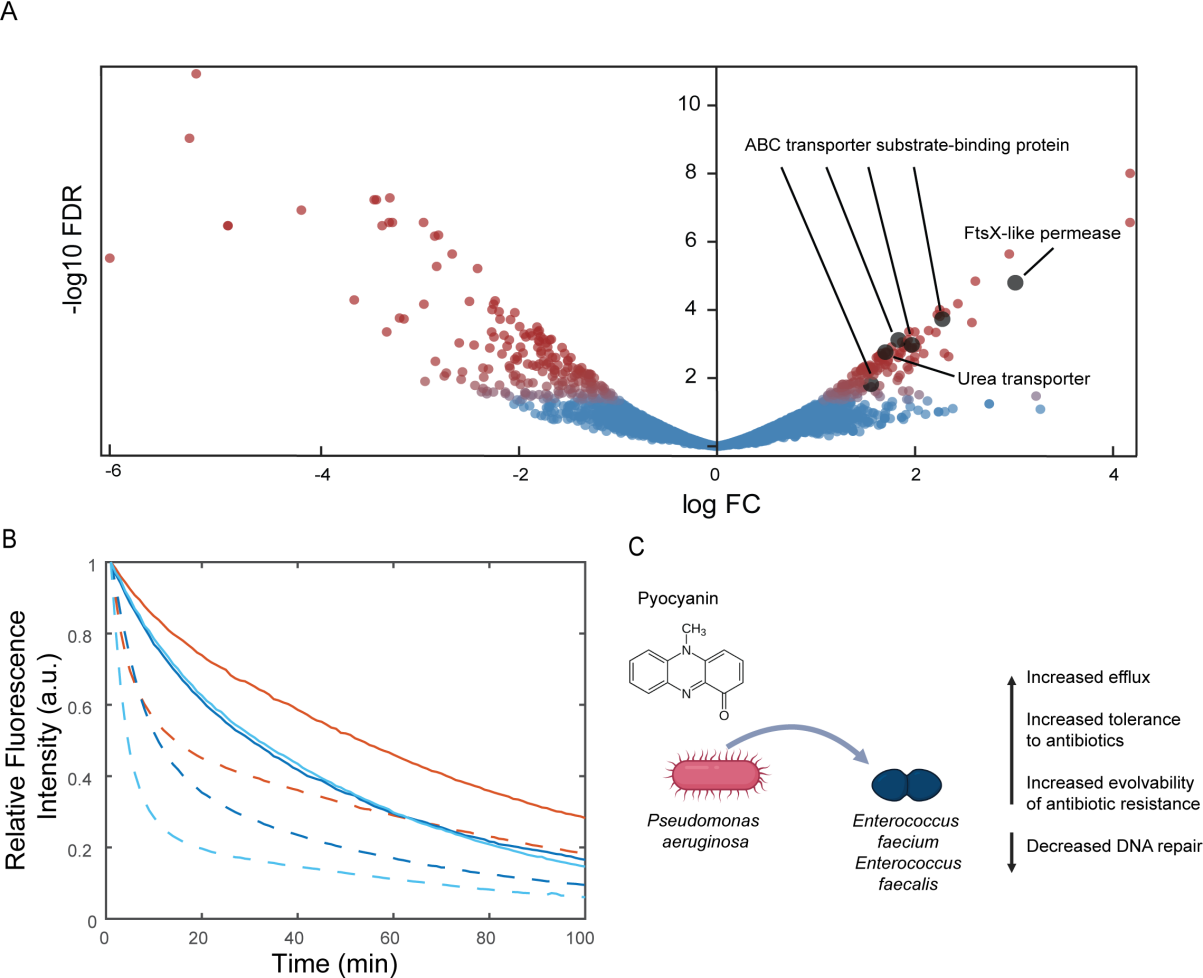
Efflux upregulated in *Enterococcus faecium* exposed to *P. aeruginosa*-conditioned medium. (**A**) Differential gene expression analysis by means of transcriptomics of *E. faecium* cultured in *P. aeruginosa* versus *E. faecium* conditioned medium shows the upregulation of several transporters. Red dots represent significantly differently expressed genes, defined as ≥ 1.5 log_2_ fold change (FC), and < 0.05 false discovery rate (FDR). Several transporters (black dots) are upregulated in the presence of conditioned medium of *P. aeruginosa* compared to the presence of *E. faecium* (control) conditioned medium. The most highly upregulated transporter is denoted as ABC transporter MacB Fts-X-like permease. (**B**) Ethidium bromide efflux assay of *E. faecium* in the presence of *P. aeruginosa*-conditioned medium, pyocyanin, and LB control medium. Bacterial efflux is determined by a decrease of the ethidium bromide fluorescence level. Overnight exposure of *E. faecium* to *P. aeruginosa*-conditioned medium (dark blue solid line) and pyocyanin (5 µg/mL, light blue solid line) increased efflux in *E. faecium* compared to the LB reference medium (red solid line). Because efflux is an active process that requires energy, the presence of glucose, an energy source (dashed lines), promotes efflux. Efflux is higher in cultures that were overnight exposed to pyocyanin (5 µg/mL, light blue dashed line) and *P. aeruginosa*-conditioned medium (dark blue dashed line) compared to the reference medium (LB, red dashed line). (**C**) Schematic depiction of pyocyanin produced by *P. aeruginosa* affecting the antibiotic resistance evolvability of enterococci.

To investigate the mechanistic basis underlying this lasting legacy effect on the evolvability of antibiotic resistance, we compared the gene expression levels of *E. faecium* exposed to *E. faecium* versus *P. aeruginosa*-conditioned medium. The results showed that under the influence of *P. aeruginosa,* several efflux pumps and transport systems displayed increased expression (Fig. 3A and Fig. S4 and S5 and Table S5 and File S1). Particularly, ABC transporter MacB Fts-X-like permease is upregulated. Genes in this complex have previously been implicated to be involved in drug tolerance via the efflux of antibiotics, particularly macrolides and cephalosporins, but also colistin and bacitracin (60, 61).

To investigate whether the increased gene expression of efflux-related transporters translated in an upregulated efflux phenotype, we performed an efflux assay in which we exposed *E. faecium* cells to either *P. aeruginosa*-conditioned medium, pyocyanin, or reference LB medium. This showed that overnight exposure to the conditioned media of *P. aeruginosa,* as well as to a physiologically relevant concentration of pyocyanin in LB, leads to increased efflux relative to reference LB medium (Fig. 3B and Fig. S6). Therefore, we conclude that pyocyanin produced by *P. aeruginosa* leads to increased efflux and increases tolerance to antibiotics.

The increased antibiotic tolerance and survival create potential for increased antibiotic-resistance evolvability via the selection of resistance mutations (62, 63). The upregulation of efflux pumps and concomitant downregulation of DNA repair and protection mechanisms has previously been implicated in increased evolution of antibiotic resistance evolution (64), which explains the higher numbers of rifampicin-tolerant *E. faecium* colonies in the evolvability assay after the transient exposure to *P. aeruginosa*-conditioned medium (Fig. 1 and 2). Together, this indicates that the transient exposure of enterococci to conditioned medium of *P. aeruginosa* containing pyocyanin leads to the increased tolerance, which affects the growth and population size, and a concomitant increase in mutation supply under antibiotic conditions due to increased efflux of antibiotics, which in turn leads to the increased evolvability of antimicrobial resistance (Fig. 3C).

## DISCUSSION

Here, we show that the transient exposure of the Gram-positive enterococci to Gram-negative *P. aeruginosa*-conditioned medium leads to the increased evolvability of antimicrobial resistance, specifically to rifampicin and nalidixic acid, due to increased efflux induced by pyocyanin, a secondary metabolite produced by *P. aeruginosa*. Due to the increased antibiotic efflux, sustained growth on the antibiotic-containing agar plates is allowed, which increases the mutation supply rate, leading to an increased evolvability of antibiotic resistance. Overexpression of efflux pump genes has been associated with antibiotic resistance in various species (31, 65, 66). Additionally, the increased expression of efflux has been associated with the relatively fast evolution of antibiotic resistance (67), and ABC transporters, such as the MacB-FtSX-like permease, have previously been implicated in increased resistance toward antibiotics, such as vancomycin (68). We show that efflux can be upregulated due to the presence of interspecies signals, such as pyocyanin.

The intracellular accumulation of pyocyanin has previously been implicated in the induced tolerance to several antibiotics in *P. aeruginosa* (69). Additionally, pyocyanin has been shown to increase SoxR-regulated efflux in the Gram-negative *P. aeruginosa* (70). Previous studies also showed that pyocyanin affected the tolerance in another Gram-negative species, *Stenotrophomonas maltophilia,* and the evolution of resistance to structurally similar synthetic antibiotics in species of the *Burkholderia cepacian* complex, which are also present in lung infections (71). Here, we report the interspecies signaling effect of pyocyanin, leading to increased efflux activity, reduced sensitivity to antibiotics, and concomitant increased antibiotic resistance evolvability for two different antibiotics, such as structurally dissimilar rifampicin and nalidixic acid, in enterococci. This study therefore extends the induction of tolerance and the promotion of antibiotic resistance by pyocyanin to Gram-positive bacteria.

Other examples with a demonstrated effect on efflux-inducing secondary metabolites are redox-active compounds, such as toxoflavin and salicylate, which are produced by *Burkholderia species* and mediate tolerance in other *Burkholderia species* (72, 73), as well as indole signaling in *E. coli* affecting efflux-mediated tolerance to norfloxacin in *E. coli* (10). We, therefore, anticipate that transient interactions mediated by secondary metabolites, within and across bacterial species, could be more widespread and may contribute to the mismatch between observed clinical antibiotic levels and *in vivo* treatment failure (72, 74).

We found that the increased evolvability of antibiotic resistance of enterococci due to the transient exposure to *P. aeruginosa*-conditioned medium lasted after removal of the conditioned medium, consistent with observations of substantial bacterial memory (75). We hypothesize that the upregulation of efflux pumps leads to the production and accumulation of efflux proteins that is sufficiently high to have a lasting effect on antibiotic efflux, even after several cell divisions on agar in the absence of the efflux-inducing presence of pyocyanin. Under such circumstances, cells experience longer survival and larger population sizes in the presence of antibiotics, potentiating the production and selection of antibiotic-resistant mutants with high resistance levels.

Due to the general effect of conditioned medium of *P. aeruginosa* on enterococci, we propose that future work should investigate whether other secondary metabolites have similar interspecies efflux-inducing properties promoting antimicrobial tolerance as pyocyanin, also in other Gram-positive species. For example, other phenazines that are structurally similar to pyocyanin produced by *Pseudomonas* species, such as phenazine-1-carboxylic acid and phenazine-1-carboxamide, may also be efflux-inducing. Additionally, understanding the molecular mechanism of induction of efflux pumps and the downregulation of DNA repair mechanisms is mandatory to understand the scope of this cross-species effect on antibiotic tolerance and evolvability.

Our study shows that not only current interactions but also past metabolic interactions with other bacterial species can have a lasting effect on the growth of enterococci in environments containing antibiotics. Therefore, direct interactions and lasting legacy effects have the potential to change the eco-evolutionary interactions in polymicrobial infection ecosystem formation and enhance the evolution of antimicrobial resistance.

## MATERIALS AND METHODS

### Isolates

We used isolates derived from polymicrobial urinary tract infections obtained for previous studies (1), except for *P. aeruginosa* PAO1 and its derivative quorum-sensing mutants (LasR, LasI), which were a kind gift from Ashleigh Griffin (University of Oxford).

### Media and materials

Liquid Luria Broth (LB) was created as follows: 10 g/L Bacto tryptone, 5 g/L yeast extract, and 10 g/L NaCl. 5x LB: 50 g/L bacto tryptone; 25 g/L yeast extract, no NaCl. Agar plates were made using LB with 15 g/L Technical agar #3 (Sigma) and different concentrations of antibiotics. Antibiotic testing and colony formation in evolvability assays were performed on 1× LB agar plates containing rifampicin (15 μg/mL, 50 μg/mL, 100 μg/mL) or nalidixic acid (150 μg/mL). The reference medium used to compare to conditioned medium in evolvability assays consisted of 1.5× LB including 0.01 M PIPES buffer (pH 6.5). Pyocyanin (P0046, Sigma) and phenazine (P13207, Sigma) were resolved in DMSO (5 mg/mL and 10 mg/mL, respectively).

### Co-growth *E. faecium* and *P. aeruginosa*

*E. faecium* and *P. aeruginosa* were grown in isolation in 5 mL LB overnight at 37°C and 200 rpm. Overnight cultures were diluted 1:500 and mixed together (1:1 volume ratio) in 5 mL LB, followed by growth for 24 h at 37°C under shaking conditions (200 rpm). Cell densities were determined by enumerating CFU/mL on Chromagar Orientation (Chromagar) after 24 h of growth at 37°C.

### Conditioned medium

Donor strains were grown in 500 mL Erlenmeyers in 200 mL LB under aerobic conditions for 48 h at 37°C at 200 rpm. All cultures were divided over 50 mL tubes and centrifuged for 15 min at 4,700 rpm at room temperature. Supernatant was collected, leaving the pellet as undisturbed as possible. The cultures were filtered using a 0.45 μm mesh filter attached to a vacuum pump set at 0.1–0.5 Bar. A 0.2 μm mesh filter was used to filter the cell-free supernatant for a second time. Conditioned medium was generated by mixing 0.5 fraction spent medium (cell-free supernatant), 0.1 fraction filter-sterilized 0.01 M PIPES buffer (pH 6.5), 0.1 fraction 5× LB, and 0.3 fraction 1× LB, such that in the hypothetical case that no nutrients would have been consumed in the conditioned medium, the concentration of LB would be similar to the reference medium.

### Antibiotic resistance

The level of antibiotic resistance before performing the evolvability assays was assessed by spotting 10 μL of overnight culture on LB agar plates containing the appropriate antibiotics. Resistance was determined by the highest concentration at which growth was observed on the LB agar plate.

### Evolvability assay

The high-throughput evolvability assay is based on the fluctuation assay developed by Luria and Delbrück to estimate mutation rates of bacterial strains (42). One pre-culture was made in LB and diluted in pre-warmed LB to a density of 10^−3^. Twenty-three microliters of this diluted culture were added to 23 mL of LB conditioned medium (inoculated density OD_600_ 10^−6^), LB plus pyocyanin, or LB plus phenazine. For each condition, at least two 96-well plates were filled with 200 µL of the bacteria medium mixture. These 96-well plates were incubated overnight under non-shaking conditions at 37°C. After 16 h, the plates were placed in a BMG Clariostar plate reader and mixed for 240 s at 600 rpm using double orbital shaking, after which the OD_600_ was recorded. Following mixing and OD_600_ measurement, 8 µL drops from each well were spotted on pre-warmed LB agar plates containing rifampicin or nalidixic acid (15 µg/mL and 150 µg/mL, respectively; i.e., twice the MIC concentration). Rifampicin and nalidixic acid are commonly used for microbial mutation rate assays (76–81). For Fig. 1, 288 replicate cultures (200 µL each, in three 96-well plates; each batch of 96 200 µL cultures per 96-well plate is considered one replicate, *N* = 1) in each condition were used to calculate the rate of resistance production. After 2 days of incubation at 37°C, the number of spots that had at least one antibiotic-resistant colony were counted. The evolvability measure was calculated using the p-0 method as:


$$Evolvability=\frac{\log\;\left(\frac{1}{p_{0}}\right)}{OD_{600}},\;\text{where }p_{0}=1-\frac{\text{spot count}}{96}.$$

This calculation allows for normalization of the samples according to their population size after overnight growth, as estimated by the optical density at 600 nm (OD_600_). The evolvability of isolates exposed to conditioned medium was normalized to the evolvability of isolates exposed to LB. While the fluctuation assay (and p0 method) was developed to determine mutation rates (43), we use it to determine the evolvability of antibiotic resistance, resulting from changes in either the mutation rate, enhanced phenotypic tolerance, or both.

### Evolvability assay plus washing in PBS and regrowth in LB

Evolvability assays were performed according to the methods described above, except for a few changes depending on the condition assessed. To assess the necessity of the presence of conditioned medium and the longer-term effect of the conditioned medium in the absence of that conditioned medium, we have both washed the cells in PBS buffer and regrown the cells in LB followed by washing. For each condition, three sets of three 96-well plates were filled with 200 µL of bacteria medium mixture and grown overnight at 37°C. We washed one subset (containing three 96-well plates, 288 × 200 µL of each condition) of the overnight cultures exposed to *P. aeruginosa*-conditioned or LB medium in PBS buffer before spotting the replicate cultures onto the rifampicin-containing LB agar plate. Additionally, another subset (consisting of three 96-well plates for each condition) of overnight cultures exposed to *P. aeruginosa*-conditioned or LB medium was washed in LB, followed by a 3-hour regrowth in LB (in the absence of conditioned medium), before samples were spotted onto rifampicin-containing agar plates. Cells were washed by centrifugation for 15 min at 4,700 rpm at RT, followed by resuspension in 37°C LB medium or PBS. The OD_600_ was determined after washing or regrowth. The third subset of overnight-exposed cultures to *P. aeruginosa*-conditioned or LB medium (containing three 96-well plates of each condition) was kept as a control for the execution of the experiment. Evolvability after washing with PBS buffer and regrowth in LB was calculated as described above, with the evolvability of *P. aeruginosa*-conditioned medium-derived cultures relative to those exposed to the LB reference.

### Assessment of resistance level of colonies derived from evolvability assay

The resistance level of the clones that appeared in the evolvability assay was assessed by regrowing the clones overnight in LB and spotting ~ 2 µL of the overnight culture on fresh agar plates containing rifampicin (50 µg/mL, 100 µg/mL). Isolates showing growth at the respective agar plates containing antibiotics were scored as resistant.

### Antibiotic tolerance assay

Two microliters of *E. faecium* overnight cultures were reinoculated into liquid cultures in 96-well plates for 24 h. The effect of *P. aeruginosa*-conditioned medium on the action of antibiotics was assessed by scoring the yield across a concentration gradient of rifampicin and nalidixic acid in the presence and absence of *P. aeruginosa*-conditioned medium, using LB as reference medium. Two-fold decrements and an additional no-drug control were used to determine the maximum antibiotic concentration that could sustain growth. Antibiotic tolerance interactions were scored as the difference in the number of antibiotic concentrations in which growth was observed in conditioned medium versus the LB reference. Growth of *E. faecium* was scored positive if OD_600_ exceeded 0.01 within 24 h. Antibiotics were added in the conditioned medium just before the start of the tolerance assay; i.e., they were absent during the growth of the donor from which the conditioned medium was produced.

### Ethidium bromide efflux assay

Five milliliters of *E. faecium* cultures were grown for 20 h at 37°C in three conditions: in LB, conditioned medium of *P. aeruginosa*, or LB with 5 µg/mL pyocyanin (resolved in DMSO), with shaking at 200 rpm. After determination of the cell density (OD_600_), the cultures were spun down for 10 min at 4,500 rpm. Cultures were resolved in pre-warmed PBS at a density of 0.1 OD_600_. These samples were incubated in PBS in 15 mL tubes for 1 h at 37°C in the presence of 5 µg/mL ethidium bromide. After incubation, cultures were spun down for 10 min at 4,500 rpm. The supernatant was discarded, and 1 mL fresh PBS was added. The OD_600_ was adjusted to 0.1, and 200 µL of these suspensions (of *E. faecium* cultured under these three conditions), with and without 0.4% glucose (efflux is an active process requiring energy; energy is provided in the form of glucose), were added to a 96-well plate, with four replicates for each condition. Fluorescence development was monitored in a BMG Clariostar plate reader (excitation 520 nm, emission 590 nm) (82).

### DNA extraction

Genomic DNA was isolated using a phenol/chloroform extraction based on the method of Sambrook (83), with some modifications as described. Bacterial cells were spun down, and cell pellets were washed in 250 µL PBS and resuspended in 250 µL PBS. Ten microliters of 20 mg/mL lysozyme was added and incubated for 30 min at 37°C. Fifteen microliters of 10% SDS and 5 µL of 20 mg/mL RNAse A were added, and incubation was continued at 37°C for 30 min. Fifteen microliters of Prot K (20 mg/mL) was added, and the sample was further incubated at 56°C for 30 min. Three hundred microliters of phenol:chloroform:isoamyl alcohol (Sigma-Aldrich) was added, the sample was mixed on a rotator at 25 rpm for 15 minutes and centrifuged for 10 min at 14,000 rpm. Supernatant was collected, and DNA was precipitated by the addition of 10% 3M Na-acetate and 1 mL 100% ice-cold ethanol. DNA was pelleted by centrifugation at 10,000 rpm for 5 min, and the pellet was washed twice with 300 µL 70% ethanol. The pellet was shortly dried and dissolved in 50 µL nuclease-free water. To remove residual RNA and further purify the DNA, a second RNAse treatment and double P:C:IAA clean-up were performed as follows. Thirty microliters of DNA solution was diluted by the addition of 150 µL of Tris-EDTA buffer and 2 µL of 20 mg/mL RNase A. Samples were incubated at 37°C for 1 h. Two hundred fifty microliters of phenol:chloroform:isoamyl alcohol was added, mixed, and centrifuged as described above. Two hundred microliters of the supernatant was transferred to a 2 mL phase-lock tube, 200 µL of phenol:chloroform:isoamyl alcohol was added, mixed, and centrifuged again as described above. One hundred eighty microliters of supernatant was transferred to a 1.5 mL DNA low-bind tube (Eppendorf), and 10% 3 M Na-acetate (pH 5.5) and 800 µL ice-cold 80% ethanol was added, and the tube was incubated on ice for 1 h to precipitate the DNA. DNA was collected by pelleting as described above, washed twice in 300 µL 70% ethanol, air-dried at room temperature for 2 min, and dissolved in 30 µL nuclease-free water.

### Whole-genome sequence analysis

The parental *E. faecium* strain was sequenced on the Illumina Novaseq and ONT Minion platforms by MicrobesNG (https://microbesng.com) to create a closed reference genome sequence, assembled using Unicycler and annotated using Prokka. Twenty-one random evolved isolates were isolated from rifampicin-containing agar plates that were either pre-exposed to LB or *P. aeruginosa*-conditioned medium during the evolvability assay. These isolates were also sequenced on the Illumina Novaseq platform by MicrobesNG, and reads were filtered using TrimAdapt. The resulting FastQ files were then mapped against the closed parental strain genome using BreSeq.

### RNA extraction, library preparation, and transcriptomic analysis

*E. faecium* cultures for RNA extraction were prepared by creating an overnight culture in LB broth with growth at 37°C, shaken at 200 rpm. This overnight culture was used to inoculate the different conditioned media, and *E. faecium* was allowed to grow into mid-exponential phase (OD ≈ 0.15) at 37°C, 200 rpm. Cultures were pelleted at 4,700 rpm for 15 min, and supernatants were discarded. Pellets were suspended in 0.5 mL medium and transferred to Eppendorf tubes and centrifuged again at 18,000 rpm for 5 min. After this, the supernatants were discarded, and pellets were flash frozen in liquid nitrogen for RNA extraction.

RNA was isolated using High Pure RNA Isolation Kit (Roche), with an adapted protocol for improved RNA microarray analysis. Pellets were thawed on ice and resuspended in 400 μL DEPC-treated MQ (100 µL DEPC per 100 mL solution). This solution was added to screw-cap tubes containing 0.5 g glass beads, 50 µL 10% sodium dodecyl sulfate (SDS), and 500 µL acid-phenol:chloroform (pH 4.5) (with indole-3-acetic acid [IAA], 125:24:1) (ThermoFisher AM9720). Tubes were placed in a bead beater (Mini-BeadBeater 607, BioSpec, USA), and homogenization was performed using 6 × 60 s pulses with 1 min interval on ice. Tubes were subsequently centrifuged at 10,000 rpm for 10 min at 4°C. The resulting upper phase was transferred to a fresh tube with 500 µL chloroform:IAA (24:1) and centrifuged again for 5 min at 10,000 rpm at 4°C. Five hundred microliters of the upper phase was again transferred to a new tube with 1 mL of the Roche lysis/binding buffer (4.5 M guanidine HCl, 30% Triton-X-100, 50 mM Tris, pH 6.6) and mixed well. Eight hundred microliters was transferred to the upper reservoir of the provided filter and collection tube. Tubes were centrifuged for 15 s at 8,600 rpm. Flow-through was discarded, and the remainder was transferred back to the upper reservoir of the filter. The tube was centrifuged again for 15 s at 8,600 rpm. Flow-through was discarded, and 100 mL of DNase I mix was added, containing 90 µL DNase buffer and 10 µL DNase I. Tubes were incubated for 30 min at 25°C. Five hundred microliters of wash buffer I was added, and the tubes were centrifuged at 8,600 rpm for 15 s. This was repeated with wash buffer II, and again with 200 µL wash buffer II, followed by centrifugation at maximum speed. The filter was then transferred to a sterile 1.5 mL Eppendorf tube. Fifty microliters of elution buffer was added directly on the filter in the upper reservoir and incubated for 10 min at room temperature. Tubes were centrifuged for 1 min at 8,600 rpm, and aliquots were stored at −80°C until the library preparation.

RNA concentrations were measured using a Nanodrop 2000c Spectrophotometer (Thermo Fisher Scientific, USA), and the quality of RNA was analyzed using gel electrophoresis. A 50 mL gel was created of 1% (w/vol) agarose in TAE buffer with 500 µL Bleach (Bleach is created by dissolving 1 tablet of a RECA bleach tablet in 100 mL MilliQ water). The mixture was incubated at room temperature for 5 min before heating the agarose to boiling temperature. Agarose was allowed to cool to 65°C before adding 2 mL ethidium bromide. After pouring the gel and letting it set, RNA samples were mixed with 2× RNA loading buffer. The 2× RNA loading buffer consisted of 9.5 mL formamide, 25 µL of 10% SDS, 2.5 mg bromophenol blue, 2.5 mg ethidium bromide, and 10 µL of 500 mM EDTA. The gel was run in TAE buffer at 100 V for 25 minutes.

RNA was reverse-transcribed into cDNA using the reagents from the Zymo-Seq Ribofree cDNA kit (Zymo Research). DNA was resuspended in 10 µL DNA elution buffer, and the mixture was transferred to a new PCR tube to be stored at −20°C before sending it for sequencing. Library preparation was performed with nuGEN RNA-sequencing kit. Samples were sequenced on the Illumina NexSeq 500 to generate 75 bases single-end reads (75SE) with an average read depth of 12 million reads per sample.

Differential gene expression was quantified using Kallisto (V 0.46.0) with read length set to 75 bp and standard deviation set to 5. The resulting data were analyzed using Voom/Limma in Degust (V 3.20) to identify significantly differentially transcribed genes (average log fold change of ≥1.5 and a FDR significance value of <0.05). Functional categories (COG, GO terms) were assigned to genes using eggnog-mapper (V 2).

## Data Availability

European Nucleotide Archive project PRJEB113294.

## SUPPLEMENTAL MATERIAL

Data S1 aac.00078-26-s0001.xlsx Transcriptomics data of *Enterococcus faecium* exposed to *Pseudomonas aeruginosa* conditioned medium vs *E. faecium* conditioned medium.

Supplemental material aac.00078-26-s0002.pdf Fig. S1 to S6; Tables S1 to S5.

Supplemental data legend aac.00078-26-s0003.pdf Legend for supplemental data.

ASM does not own the copyrights to Supplemental Material that may be linked to, or accessed through, an article. The authors have granted ASM a non-exclusive, world-wide license to publish the Supplemental Material files. Please contact the corresponding author directly for reuse.
