# Supplementary material for: Pyocyanin produced by Pseudomonas aeruginosa creates legacy effects that promote antibiotic resistance evolution in enterococci
Source: Antimicrob Agents Chemother. 2026 Jun 22;70(8):e00078-26. doi: 10.1128/aac.00078-26 (PMC13436219; doi:10.1128/aac.00078-26)
Supplement: Supplementary file 1 [file aac.00078-26-s0002.pdf]

SUPPLEMENTARY INFORMATION

M.G.J. de Vos, V. Jansen, O. Bouhlali, A. Vlasblom, L.E. Zandbergen, I. van der Windt, C.I. Sey, J. Kool, R. Nijland, A. de Jong, O.P. Kuipers, S. Dunn, A. McNally, J.A.G.M. de Visser

Pyocyanin produced by *Pseudomonas aeruginosa* Creates Legacy Effects That Promote Antibiotic Resistance Evolution in Enterococci

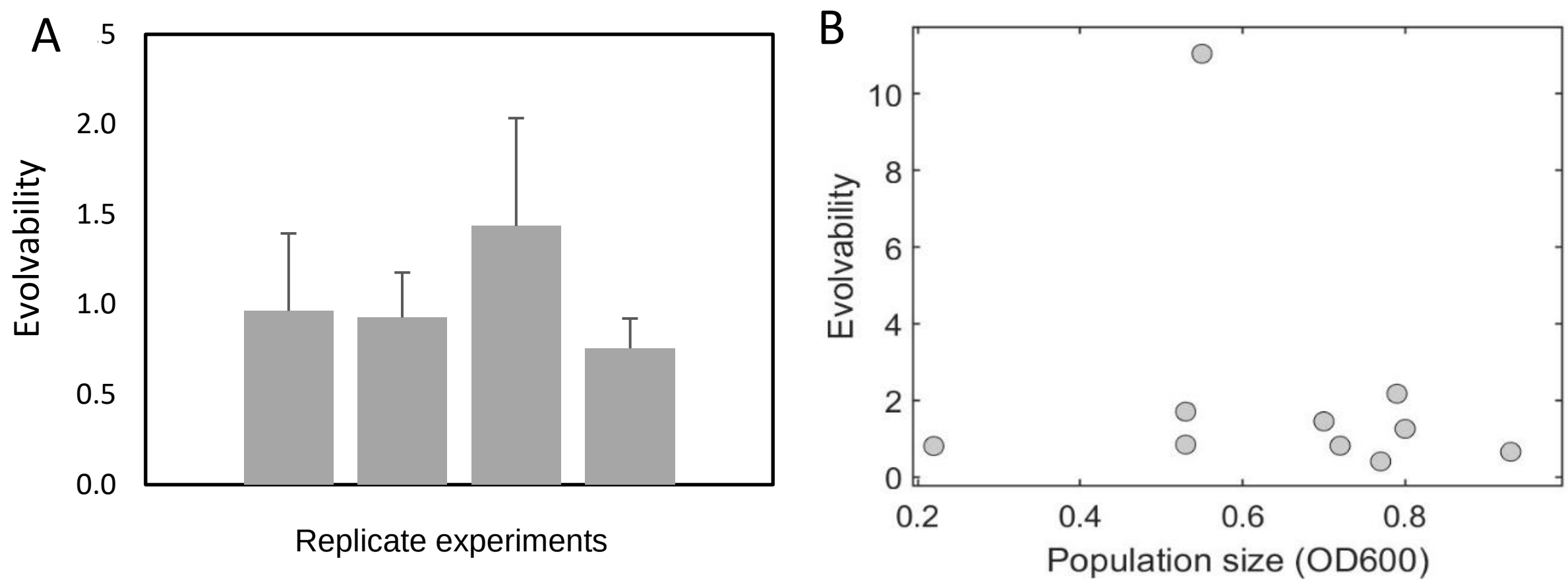

Figure S1. Evolvability of rifampicin resistance in *E. faecium* is not correlated with the level of nutrients or population size.

(a) Different nutrient level does not lead to changes in evolvability of rifampicin resistance. Evolvability, as measured by the evolvability assay, of four replicate experiments under higher nutrient conditions (1.5 x LB) normalized to evolvability under lower nutrient conditions (1 x LB) ($p > 0.05$, Student's t-test, bar graphs show averages with whiskers depicting the standard deviations, N = 3 for each experiment). **(b) No correlation between population size in conditioned medium and evolvability.** Population size versus average rifampicin evolvability of *E. faecium* grown under different conditioned medium conditions. Population size measured after 24 hours and evolvability relative to the evolvability in LB: *K. pneumoniae, E. coli* (two different isolates), *P. mirabilis* (two different isolates), *S. aureus*, *S. haemolyticus*, *E. faecium*, *E. faecalis*, and *P. aeruginosa*. Population size in the presence of conditioned medium does not correlate with evolvability of rifampicin resistance due to exposure to that conditioned medium (Materials and Methods). Only the evolvability of rifampicin resistance of *E. faecium* due to the exposure to *P. aeruginosa* conditioned medium is markedly higher (Evolvability > 10).

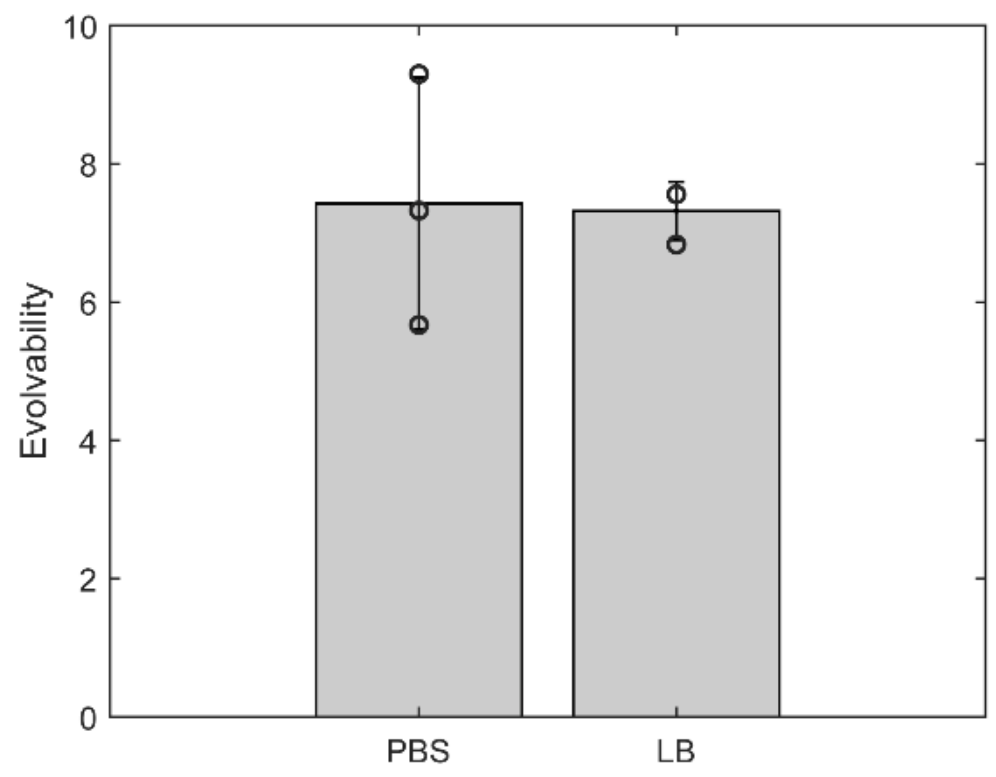

Figure S2. The legacy evolvability effect due to *P. aeruginosa*-conditioned medium overnight exposure is conserved after washing in PBS and regrowth in LB. Washing with PBS, and washing followed by regrowth in LB for three hours maintains the rifampicin resistance evolvability effect due to *P. aeruginosa* conditioned medium (Materials and Methods, Student's t-test, $p > 0.05$, N = 3 for both conditions). Bar graphs show average evolvability of rifampicin antibiotic resistance, with standard deviation. Evolvability of rifampicin resistance is measured as the rate of rifampicin resistance in populations formerly exposed to conditioned medium normalized to the rate of rifampicin resistance formerly exposed to LB reference medium.

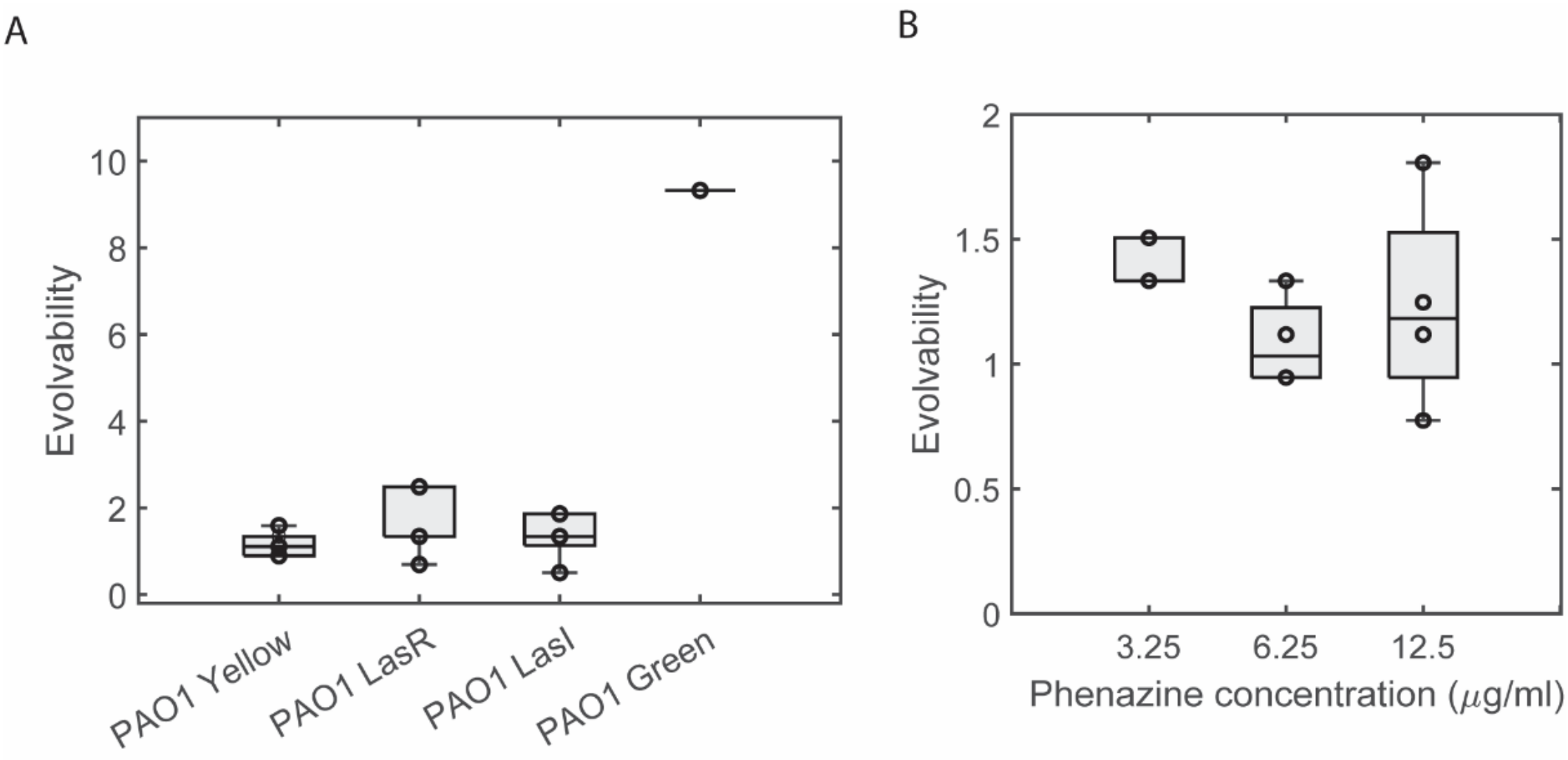

Figure S3. Increase in antibiotic resistance evolvability associated with quorum sensing of *P. aeruginosa.* (a) Increase of evolvability of antibiotic resistance by green-tinted version of PAO1 conditioned medium, not by quorum sensing mutants. Particularly the exposure to the conditioned medium of the aerobically incubated green-tinted version of *P. aeruginosa* PAO1 leads to the increase in evolvability, but also the LasR-PAO1-derived quorum sensing mutant grown under lower oxic environments, whereas conditioned medium derived from the un-tinted generated (LB-yellow) PAO1 and LasI PAO1-derived quorum sensing mutant of cultures grown under lower oxic environments do not lead to an increase in evolvability (Materials and Methods, One-way ANOVA, Dunnett's test, PAO1 Yellow p = 0.9, PAO1 LasR p = 0.02, PAO1 LasI p = 0.9, PAO1 Green p << 0.01, N = 6 for each condition). Evolvability of rifampicin resistance is measured as the rate of rifampicin resistance in conditioned medium normalized to the rate of rifampicin resistance in LB reference medium**.** **(b) Phenazine does not lead to increased evolvability.** Phenazine, a structural analogue and precursor of pyocyanin does not lead to an increased evolvability of rifampicin antibiotic resistance, as tested via the evolvability assay (Materials and Methods, One-way ANOVA, p > 0.05, N = 5, 4, 4 for each respective condition). Evolvability of rifampicin resistance is measured as the rate of rifampicin resistance in LB containing phenazine normalized to the rate of rifampicin resistance in LB reference medium**.** The lines in the box plots represent the

median, bottom and top edges represent the lower and upper quartiles, whiskers the end points of the dataset.

Figure S4. Differential gene expression of *E . faecium* in the presence of *P. aeruginosa* conditioned medium compared to in *E. faecium*-conditioned medium.

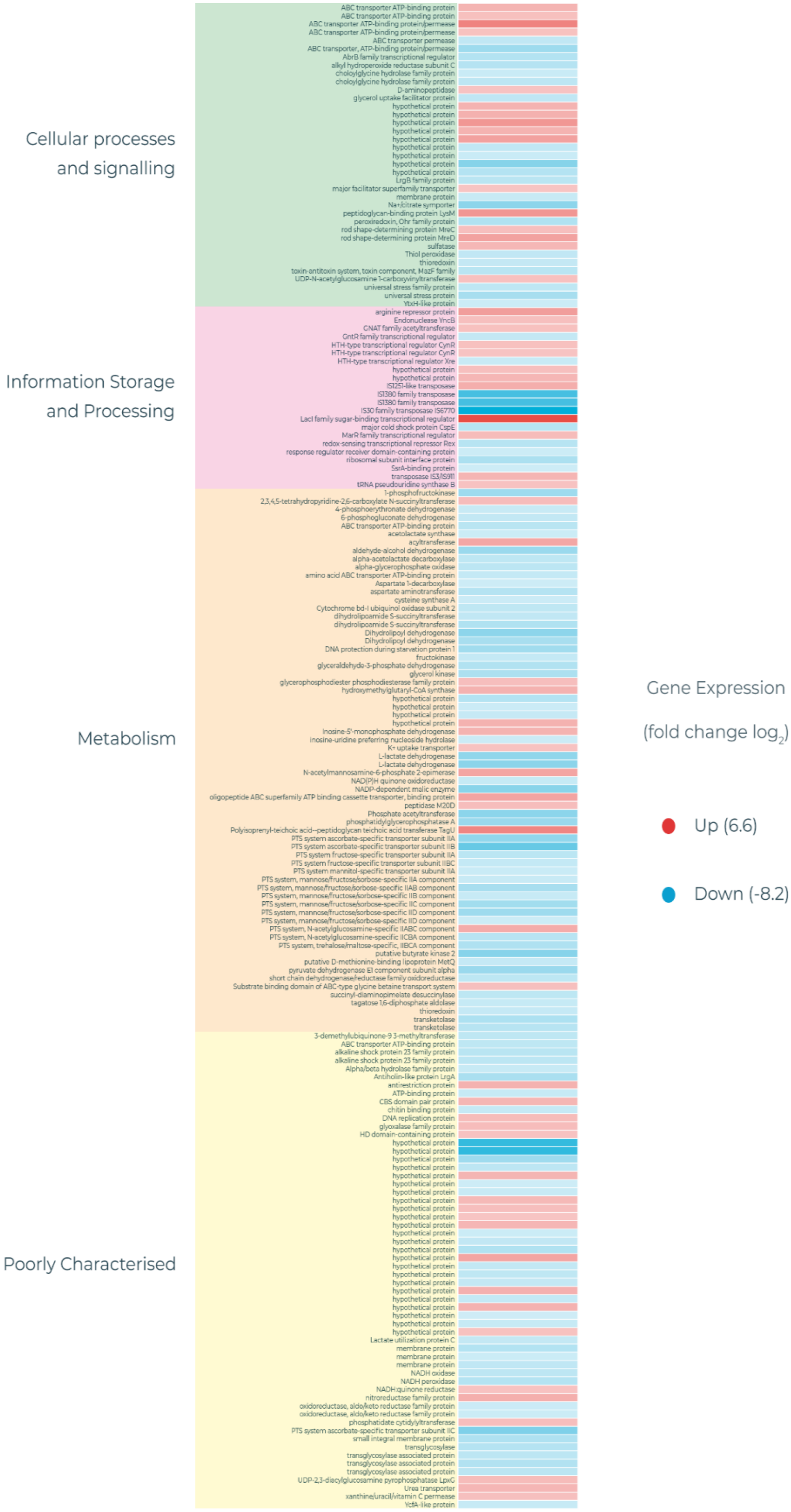

Figure S5. Enrichment analyses transcriptomics

Proportional abundance (%) of (**b**) COG categories, and (**b**) Larger organizational COG groups undergoing significant differential expression in *P. aeruginosa* versus *E. faecium* conditioned media.

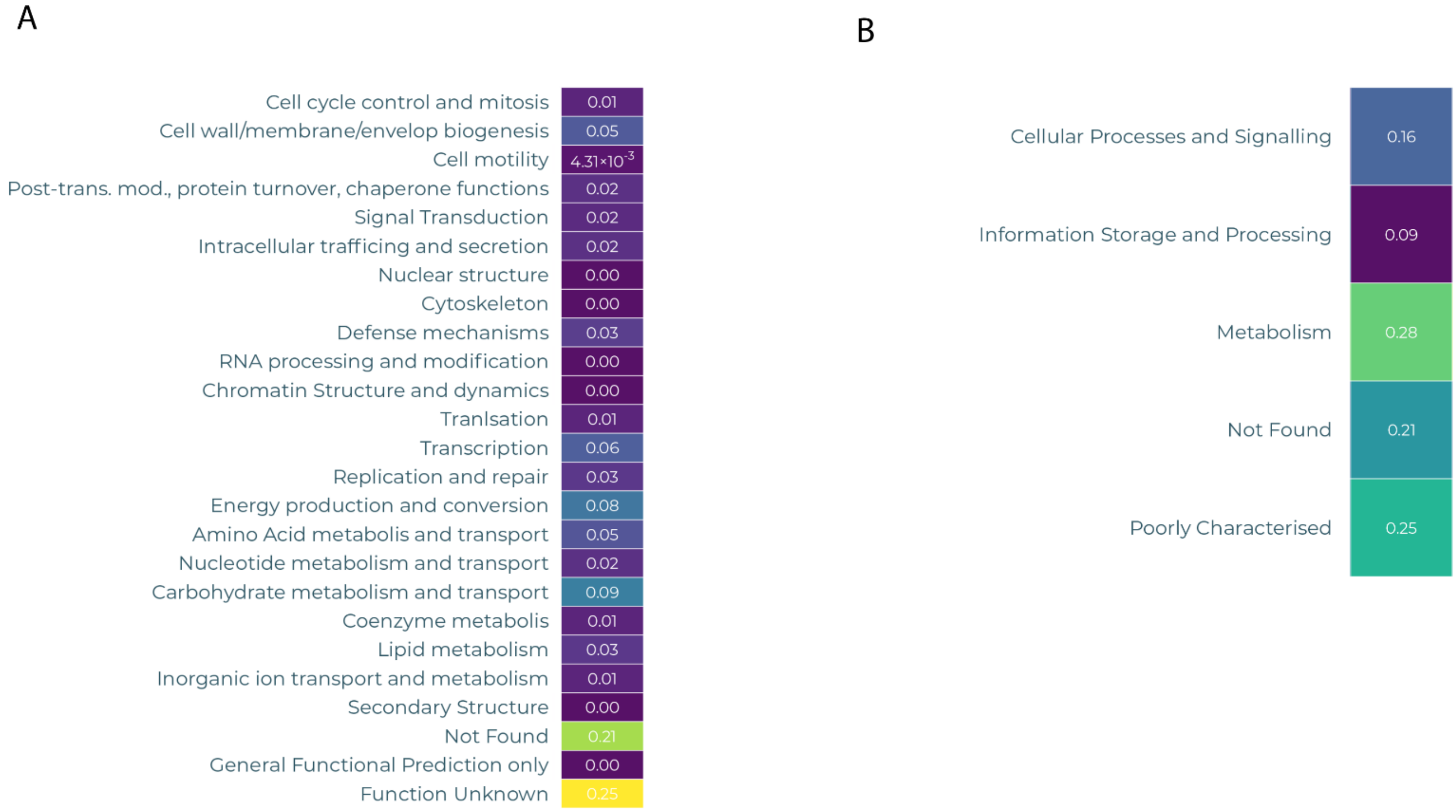

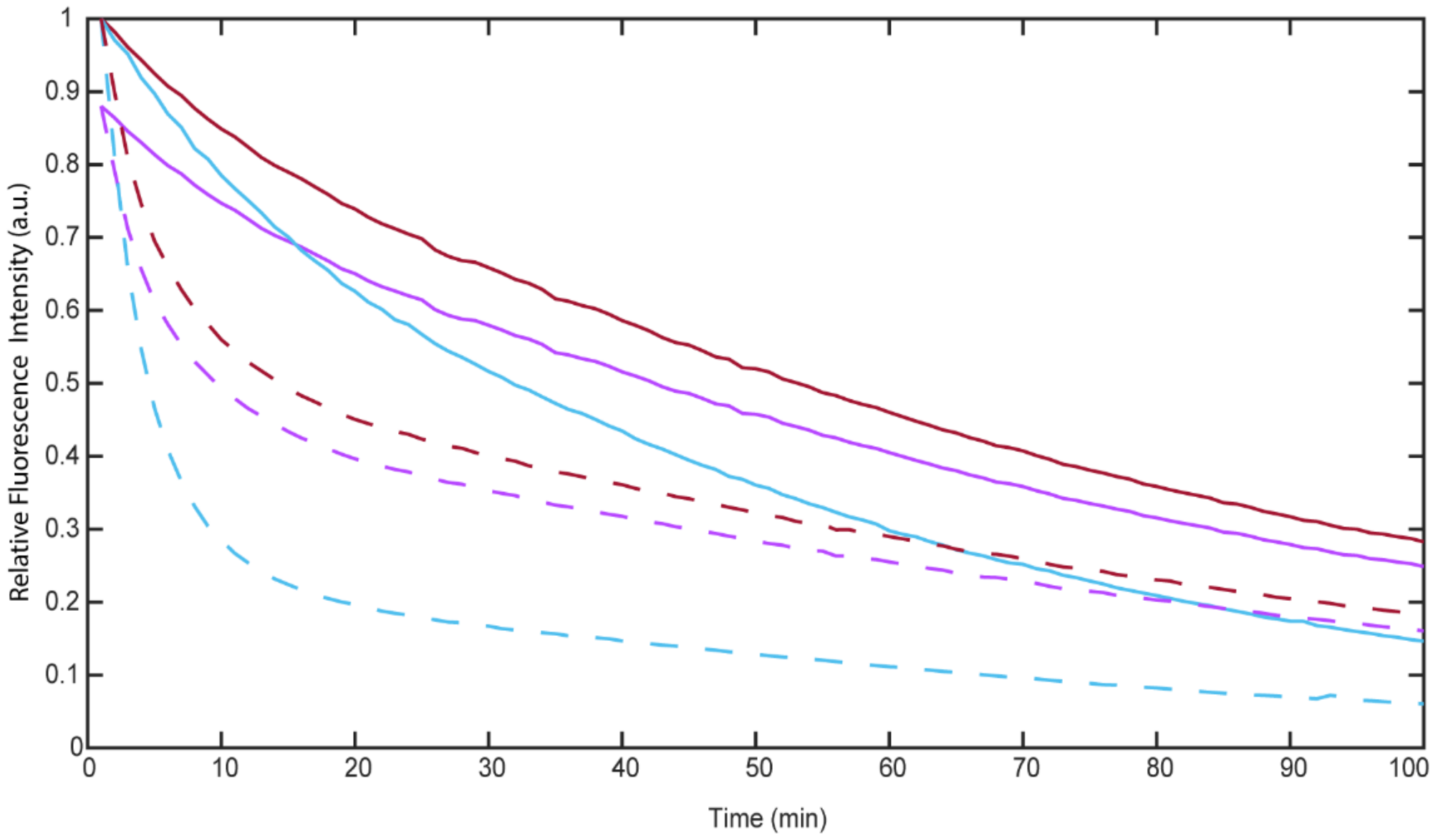

Figure S6. Ethidium bromide efflux assay. Data from Fig. 3B and simulated data to investigate whether the lower fluorescence of ethidium bromide due to competitive binding with pyocyanin to the DNA of *E. faecium* can account for the fast decrease of fluorescence. Red, measured data of efflux assay exposed to LB only (line without glucose, dashed line with glucose, Fig. 3B). Light blue, measured data of efflux assay exposed to 5 µg/mL pyocyanin (line without glucose, dashed line with glucose, Fig. 3B). Purple, simulated data; 12% lower fluorescence compared to measured data exposed to LB (reference), due to potential competitive binding of pyocyanin and ethidium bromide to DNA (line, LB data minus 12% fluorescence without glucose; dashed line, LB data minus 12% fluorescence with glucose).

Ethidium bromide is assumed to compete with pyocyanin for DNA binding[1,2], which potentially leads to lower fluorescence levels in the ethidium bromide efflux assay, due to residual levels of pyocyanin present after washing with PBS. Indeed, at the start of the efflux assays, the absolute level of fluorescence of cultures overnight exposed to of 5 µg/mL pyocyanin followed by a wash in PBS, was 12% lower compared to the level of fluorescence in the reference cultures (overnight exposure of LB only), for cultures derived from OD600=0.1. To investigate whether that 12% lower fluorescence level, would have a similar effect as observed for cultures

previously exposed to pyocyanin, we simulated a condition with a 12% lower fluorescence level over the course of the efflux assay, compared to the measured reference data (purple lines, LB). We observe that the dynamics do not come close to the much stronger effect on efflux-mediated ethidium bromide-fluorescence of cultures previously exposed to pyocyanin. We, therefore, conclude that cultures previously exposed to pyocyanin experience reduced ethidium bromide fluorescence beyond its effect through quenching.

Table S1. Cell densities from overnight co-cultures of *E. faecium* and *P. aeruginosa* in LB.

	E. faecium CFU/ml	*P. aeruginosa* CFU/ml
Replicate 1	**$7.5*10^{6}$**	**$4.7*10^{8}$**
Replicate 2	**$7.5*10^{6}$**	**$5.88*10^{8}$**
Replicate 3	**$1*10^{7}$**	**$5.45*10^{8}$**

Table S2. pH differences of the spent medium fraction of the conditioned medium, in the absence of 0.1 buffer fraction. In the presence of the buffer the pH is ~6.5.

	pH
P. aeruginosa spent medium (cell free extract)	**7.5**
E. coli spent medium (cell free extract)	**7**
E. faecium spent medium (cell free extract)	**7**

Table S3. Different and fewer *rpoB* mutations generated under the transient exposure of *P. aeruginosa*-conditioned medium compared to LB medium.

rpoB mutations of rifampicin resistant clones derived from the phenotypic evolvability assay, after overnight exposure to LB or *P. aeruginosa* conditioned medium. After overnight exposure to LB frequently-observed *rpoB* mutations are found in rifampicin resistant clones, such as H486Y, S491L and G482D [3]. After overnight exposure to *P. aeruginosa*, *rpoB* mutations that differ from those typical *rpoB* mutations are found in rifampicin resistant clones. Moreover, fewer *rpoB* mutations were found in clones that were previously exposed to *P. aeruginosa* (5 of 23 mutations), compared to those exposed to LB (9 of 18 mutations, Fisher's $p = 0.0464$).

**Conditioned medium**<br>***P. aeruginosa***	**LB**
Q473K (CAG→AAG)	S491L (TCA→TTA)
Q473K (CAG→AAG)	S491L (TCA→TTA)
H486L (CAT→CTT)	S491L (TCA→TTA)
H486D (CAT→GAT)	H486Y (CAT→TAT)
G482D (GGT→GAT)	H486Y (CAT→TAT)
	H486Y (CAT→TAT)
	H486Y (CAT→TAT)
	H486Y (CAT→TAT)
	G482D (GGT→GAT)

Table S4. Mutations other than in *rpoB* in mutants derived from evolvability assay.

Mutations in other genes than *rpoB* in rifampicin resistant clones derived from the phenotypic evolvability assay, after overnight exposure to LB or *P. aeruginosa*-conditioned medium. Other mutations were found in clones that were previously exposed to *P. aeruginosa*, compared to those exposed to LB. The latter mostly had *rpoB* mutations that are often found in rifampicin-resistant mutants [3], Table S3.

***P. aeruginosa* conditioned medium**		**LB reference medium**	
pdhD	Dihydrolipoyl dehydrogenase	*L_02668* →	hypothetical protein
ftsA_1	Cell division protein FtsA	*yfkN_2* ←	Trifunctional nucleotide phosphoesterase protein YfkN
yfkN_2 ← / ← *glmU*	Trifunctional nucleotide phosphoesterase protein YfkN/Bifunctional protein GlmU	*eutB* ←	Ethanolamine ammonia-lyase heavy chain
pyrF	Orotidine 5'-phosphate decarboxylase	*lytR_1* ←	Transcriptional regulator LytR
nrnA	putative bifunctional oligoribonuclease and	*pbpF_1* ←	Penicillin-binding protein 1F

	PAP phosphatase NrnA (2 clones)		
carA	Carbamoyl-phosphate synthase small chain	*L_03056* →	hypothetical protein
sugC_1 ← / → *mgsA*	Trehalose import ATP-binding protein SugC/Methylglyoxal synthase	*L_01950* →	hypothetical protein
L_02631	putative ABC transporter ATP-binding protein	*L_00734* →	hypothetical protein
helD ← / → *ycfH*	Helicase IV/putative metal-dependent hydrolase YcfH (2 clones)		
stp	Multidrug resistance protein Stp		
yfkN_2	Trifunctional nucleotide phosphoesterase protein YfkN		
L_00636 ← / → *L_00637*	hypothetical protein/hypothetical protein		
L_03056 →	hypothetical protein		

L_02442 ←	hypothetical protein		

Table S5. List of the ten most overexpressed genes of *E. faecium* exposed to *P. aeruginosa*-conditioned medium versus *E. faecium* exposed to *E. faecium*-conditioned medium, assessed via transcriptomics.

Gene function	Log2-fold change	FDR	p-value
LacI family sugar-binding transcriptional regulator	4.17	1.04e-8	1.02e-11
Polyisoprenyl-teichoic acid--peptidoglycan teichoic acid transferase TagU	2.95	2.38e-6	1.40e-8
ABC transporter ATP-binding protein/permease MacB FtsX-like permease family	3.01	1.64-e5	12.4e-7
Rod shape-determining protein MreD	2.31	1.24e-4	1.33e-6
Oligopeptide ABC superfamily ATP binding cassette transporter, binding protein OppA	2.28	1.91e-4	2.44e-6
ABC transporter ATP-binding protein	1.91	9.84e-4	2.32e-5
Peptidoglycan-binding protein LysM	2.61	1.48e-5	1.06e-7
PTS system, N-acetylglucosamine-specific IIABC component ptsG PTS_EIIB,PTS_EIIC	2.09	1.94e-3	6.37e-5
ABC transporter ATP-binding protein	1.61	2.53e-3	9.15e-5
CBS domain pair protein YkuL	1.86	1.90e-3	5.91e-5
MarR family transcriptional regulator	1.72	1.81e-3	5.39e-5

References

1. Das, T. *et al.* Phenazine virulence factor binding to extracellular DNA is important for Pseudomonas aeruginosa biofilm formation. *Scientific Reports 2015 5:1* **5**, 8398- (2015).
2. Das, T., Kutty, S. K., Kumar, N. & Manefield, M. Pyocyanin Facilitates Extracellular DNA Binding to Pseudomonas aeruginosa Influencing Cell Surface Properties and Aggregation. *PLoS One* **8**, e58299 (2013).
3. Enne, V. I., Delsol, A. A., Roe, J. M. & Bennett, P. M. Rifampicin resistance and its fitness cost in Enterococcus faecium. *J. Antimicrob. Chemother.* **53**, 203–207 (2004).
