# Supplementary material for: Pyocyanin produced by Pseudomonas aeruginosa creates legacy effects that promote antibiotic resistance evolution in enterococci
Source: Antimicrob Agents Chemother. 2026 Jun 22;70(8):e00078-26. doi: 10.1128/aac.00078-26 (PMC13436219; doi:10.1128/aac.00078-26)
Supplement: Supplementary file 2 [file aac.00078-26-s0003.pdf]

Supplementary Data 1

Transcriptomics data of *Enterococcus faecium* exposed to *Pseudomonas aeruginosa* conditioned medium versus *E. faecium* conditioned medium.
